# Outpatient-Based Therapy of Oral Fludarabine and Subcutaneous Alemtuzumab for Asian Patients with Relapsed/Refractory Chronic Lymphocytic Leukemia

**DOI:** 10.1155/2009/547582

**Published:** 2008-02-25

**Authors:** William Y. K. Hwang, Claire Dearden, Yvonne S. M. Loh, Yeh C. Linn, Sim L. Tien, Gerrard K. H. Teoh, Gee F. How, Kee K. Heng, Yeow T. Goh, Lai H. Lee

**Affiliations:** ^1^Department of Hematology, Singapore General Hospital, Singapore 169608; ^2^Cancer and Stem Cell Biology, Duke-NUS Graduate Medical School, Singapore 169547; ^3^CLL Unit, Royal Marsden Hospital, London SW3 6JJ, UK

## Abstract

*Background*. Intravenous alemtuzumab and fludarabine are effective in combination for the treatment of chronic lymphocytic leukemia (CLL), but require hospital visits for intravenous injection. We performed a pilot study to assess the safety and efficacy of outpatient-based oral fludarabine with subcutaneous alemtuzumab (OFSA) for the treatment of relapsed/refractory CLL. *Results*. Depending on their response, patients were given two to six 28-day cycles of subcutaneous alemtuzumab 30 mg on days 1,3, and 5 and oral fludarabine 40 mg/m^2^/day for 5 days. Median patient age was 74. The lymphocyte counts of all five patients fell after the 1st cycle of treatment and reached normal/low levels on completion of 2 to 6 cycles of therapy. Platelet counts and hemoglobin were unaffected. All five patients achieved complete hematological remission, while two attained minimal residual disease negativity on 4-color flow cytometry. *Conclusions*. Our OFSA regimen was effective in elderly Asian patients with relapsed/refractory CLL, and it should be investigated further.

## 1. Background

Chronic lymphoproliferative disorders are considered rare in Asian countries. A recent 3-year study of 342 consecutive leukemia patients from 2 regional hospitals in Hong Kong, however, showed that (CLL) was diagnosed in 19% of Chinese patients with leukemia [1]. Alemtuzumab has just been approved as a single agent for first-line therapy of chronic lymphocytic leukemia [2]. Intravenous alemtuzumab administered in combination with fludarabine has been shown to be an effective chemoimmunotherapy regimen in CLL, even among patients with relapsed or refractory disease who were unresponsive to prior therapy with either agent alone [3, 4]. However, both these drugs had to be given intravenously. Subcutaneous (SC) administration of alemtuzumab can reduce the occurrence of acute infusion-related reactions while maintaining therapeutic efficacy comparable to that of intravenous alemtuzumab [5, 6]. Moreover, oral fludarabine has been made available, and is found to be as effective as the intravenous combination even in combination therapy [7]. Based on these findings, we conducted a single arm pilot clinical study to study the safety and efficacy of subcutaneous alemtuzumab in combination with oral fludarabine for the treatment of CLL, in order to provide patients with a self-administered outpatient-based regimen for treatment of relapsed/recurrent CLL.

## 2. Patients and Methods

**Figure 1:**
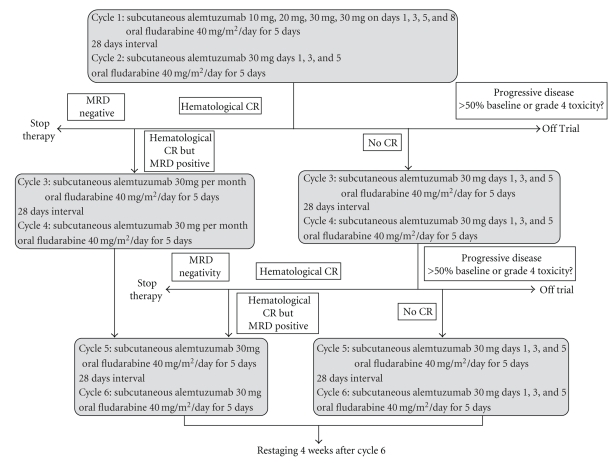
OFSA Treatment schedule. CR: Complete remission, MRD: Minimal residual disease.

Patients with relapsed or refractory CLL with failure of at least one prior chemotherapy regimen were eligible to enroll in this study, which was approved by the Institutional Review Board of the Singapore General Hospital. Response to therapy was assessed based on the National Cancer Institute Working Group criteria (NCI-WG) [8]. Evaluation of B cell clonality and minimal residual disease (MRD) was performed on bone marrow specimens by both DNA methods (consensus immunoglobulin heavy-chain polymerase chain reaction) [9] as well as flow cytometry (estimated sensitivity of 0.01%) [10] after every 2 cycles of treatment. Oral fludarabine and subcutaneous alemtuzumab (OFSA) chemotherapy comprised induction and consolidation cycles as outlined in Figure 1 and were given in 28 day cycles, whenever feasible. In the first induction cycle, patients were given self-administered SC alemtuzumab 10 mg, 20 mg, 30 mg and 30 mg in dose escalation on days 1, 3, 5 and 8, respectively, together with oral fludarabine 40 mg/m^2^/day for 5 days of each 28-day cycle. For cycle 2 of induction, SC alemtuzumab 30 mg was given three times on days 1, 3, and 5 together with oral fludarabine 40 mg/m^2^/day for 5 days of each cycle. Patients were premedicated with steroids (100 mg hydrocortisone) just before the first dose of alemtuzumab of the first cycle. Depending upon response, patients received 2 to 6 cycles of induction: if complete remission (CR) was attained without MRD negativity on four-color flow cytometry and IgH clonality assays, consolidation therapy was given comprising SC alemtuzumab 30 mg on day 1 and oral fludarabine 40 mg/m^2^/day for 5 days of each cycle. Therapy was discontinued if molecular CR with MRD negativity was attained. No more than 6 cycles of therapy, whether for induction or consolidation, were administered (Figure 1). Patients were given oral cotrimoxazole and acyclovir prophylaxis. Cytomegalovirus (CMV) antigen (Ag) titers were assayed for patients with fever and all blood products were irradiated and filtered. Oral paracetamol 1 g and chlorpheniramine were given before alemtuzumab injections. IV hydrocortisone 100 mg was administered before test dose administration during the first induction cycle; with monitoring of blood pressure, heart rate and temperature were performed every 15 minutes for 1 hour after.

## 3. Results

### 3.1. Patient Characteristics

As shown on Table 1, patients were all males with median age of 72 years (range, 60–81 years), some with other comorbidities. All 5 patients had received at least 2 prior lines of therapy, 4 of whom had received fludarabine alone or in combination with other chemotherapy. Recruitment was limited to five patients by the funding available for this pilot study. The CLL-FISH panel revealed 11q23 (consistent with ATM gene deletion) and trisomy 12 abnormalities in 2 patients; in both cases, trisomy 12 was also revealed using conventional cytogenetics, with additional rearrangement of chromosome 13 in one patient and multiple structural abnormalities in the other (Table 1). The FISH panel also revealed 17p13.1 deletion (consistent with p53 deletion) in one patient, 13q14 deletion in one patient, whereas one patient had no chromosomal aberrations. In the latter patient with no detectable chromosomal abnormalities by FISH, conventional cytogenetics revealed translocations between chromosomes 13 and 17. FISH analysis was used for prognostication and not for measurement of residual disease or clonal evolution and was not repeated after completion of therapy.

### 3.2. Response to Therapy

All 5 patients responded to therapy; 2 patients achieved a partial response (PR), 2 had an MRD-negative CR and one had an MRD-positive CR (Table 1). Patient no. 1 received 4 cycles of induction followed by 2 cycles of consolidation therapy and subsequently achieved a MRD-positive CR. Patients no. 2 and no. 3 achieved a MRD-negative CR after completing only 2 cycles of induction therapy, and treatment was therefore halted. Patient no. 4 was lost to follow-up after 2 cycles of induction therapy and did not complete therapy. However, he returned to the clinic 6 months later and had relapsed, whereupon he was given melphalan therapy, to which he did not respond. OFSA combination therapy was then reinitiated off-protocol, leading the patient to achieve an MRD-positive CR. Patient no. 5 achieved clearance of blood and marrow lymphocytosis albeit the persistence of a palpably enlarged spleen after 6 cycles of induction chemotherapy. He remained MRD positive. He did not receive consolidation as the trial allowed only for 6 cycles of therapy. The median duration of follow-up for the 5 patients was 11 months (range, 8–13 months).

### 3.3. Leukocyte Response and Side Effects

The mean lymphocyte counts of all patients fell from a mean baseline value of 63,610/*μ*L to 42,610/*μ*L after the first cycle of treatment and further decreased to 1,500/*μ*L after completion of 2 to 6 cycles of therapy. Two patients developed neutropenia, as defined by an absolute neutrophil count of less than 1,000/*μ*L (one of whom had a neutrophil count <500/*μ*L), but both recovered promptly after granulocyte-colony stimulating factor (G-CSF) administration. The platelet counts (mean 114,000/*μ*L prior to and 111,400/*μ*L after therapy) and hemoglobin levels (mean 11.7 g/dL before and 11.6 g/dL after therapy) remained unaffected, and no patient required platelet or red cell transfusion support during the study. CMV reactivation did not occur, but 2 patients developed rapidly reversible neutropenic sepsis requiring empiric antibiotics and transient administration of G-CSF. More than 4 months after completing the trial, Patient no. 1 had recurrence of peripheral blood prolymphocytosis and disease-related cytopenias with concurrent chryseobacterium meningosepticum infection of the blood, to which he finally succumbed. Excluding the initial admission, none of the patients required hospitalization during the period of the trial, except for Patient no. 1 and Patient no. 4 who were hospitalized for concomitant infection and recurrence of disease 4 to 6 months after completion of therapy.

## 4. Discussion

There are several noteworthy observations from this study. Four of the 5 patients responded to this combination regimen despite failing previous fludarabine treatment and 2 attained a MRD negative status. Importantly, this combination regimen was capable of inducing responses (1 CR, 1 MRD-negative CR, 1 PR) even in patients with poor-risk cytogenetic abnormalities such as 11q and 17p deletions; this finding is consistent with recent reports from the UK CLL02 study of SC alemtuzumab with or without oral fludarabine in patients with relapsed/refractory CLL [11]. Although no preemptive therapy or CMV prophylaxis in the form of IV ganciclovir or oral valganciclovir was given, symptomatic CMV reactivation was not observed. This could be because of small patient numbers. Overall, the SC alemtuzumab and oral fludarabine combination regimen was well tolerated and induced promising responses in elderly patients with relapsed CLL, including achievement of MRD negativity in 2 patients. Importantly, this regimen provided elderly Asian patients (four of whom were more than 70 years of age) with a reasonably well-tolerated, self-administered, outpatient-based treatment of relapsed/recurrent CLL. With a median age of patients' population of 74 years (range, 60–81 years), this report provides evidence that the association of fludarabine and alemtuzumab is feasible also in a select group of elderly Asian patients. These responses are also comparable to those observed with patients from Western countries, who are treated with alemtuzumab and fludarabine combinations. In light of these encouraging results, the OFSA regimen should be extended to larger multicenter clinical trials.

**Competing Interests**

CD is a paid speaker and consultant for Bayer-Schering. WH has received research funding for this study from Bayer-Schering. The clinical trial was supported by a research grant from Schering AG. The other authors have no competing interests to declare.

**Authors' Contributions**

WH conceived of the study, coordinated in its design and drafted the manuscript. CD participated in manuscript revisions. YSL, YCL, and TSL contributed patients and participated in coordination of the study. GKT participated in design of the study. LHL provided helpful advice and facilitated the conduct of the study. All authors read and approved the final manuscript.

## Figures and Tables

**Table 1:** Patient characteristics, treatment duration, and response.

Parameters	Patient no. 1	Patient no. 2	Patient no. 3	Patient no. 4	Patient no. 5
Sex	Male	Male	Male	Male	Male

Age, years	72	70	81	60	74

Comorbidities		Ureteric stone	Benign prostatic hypertrophy	Thyroiditis, hepatitis B carrier	Diabetes mellitus, hypt stomach ulcer, hernia operation

Previous chemotherapy	CVP, FC	CP, FC	CVP, F	CP	C, F

FISH abnormalities	(Del)11q22.3 (deletion of *ATM*), trisomy 12	None	(Del)11q22.3 (deletion of *ATM*), trisomy 12	(Del)13q14 (deletion of *RB1* and D13S25)	(Del)17p13.1 (deletion of *p53*)

Conventional (metaphase) cytogenetics analysis	Numerous structural rearrangements in 7 of 20 cells; trisomy 12 in 5 other cells	t(13;17); loss of Y chromosome in 5 other cells	Trisomy 12; structural rearrangement on chromosome 13 in 1 of 20 cells	Loss of Y chromosome in 3 cells	Complex structural rearrangements in 2 of 18 cells

No. of induction cycles	4	2	2	2*	6

No. of consolidation cycles	2	0	0	0	0

Response	CR	CR	CR	PR*	PR

MRD-negativity	No	Yes	Yes	No	No

C = cyclophosphamide; CP = chlorambucil and prednisolone; CR = complete response; CVP = cyclophosphamide, vincristine and prednisolone; F = fludarabine; FC = fludarabine and cyclophosphamide; FISH = fluorescence in situ hybridization; MRD = minimal residual disease; PR = partial response.

*Patient was lost to follow-up and did not complete protocol-specified therapy.
